# Comprehensive Lifestyle-Modification in Patients with Ulcerative Colitis–A Randomized Controlled Trial

**DOI:** 10.3390/jcm9103087

**Published:** 2020-09-24

**Authors:** Jost Langhorst, Margarita Schöls, Zehra Cinar, Ronja Eilert, Kerstin Kofink, Anna Paul, Christina Zempel, Sigrid Elsenbruch, Romy Lauche, Mohamed Ahmed, Dirk Haller, Holger Cramer, Gustav Dobos, Anna K. Koch

**Affiliations:** 1 Department of Internal and Integrative Medicine, Sozialstiftung Bamberg, Germany, Chair for Integrative Medicine, University of Duisburg-Essen, Buger Straße 80, 96049 Bamberg, Germany; 2 Integrative Gastroenterology, University of Duisburg-Essen, Evang. Kliniken-Essen-Mitte, Am Deimelsberg 34 a, 45276 Essen, Germany; margarita_schoels@web.de (M.S.); zehra-cinar@gmx.de (Z.C.); r.m.eilert@web.de (R.E.); kerstinkofink@gmx.de (K.K.); 3 Department of Internal and Integrative Medicine, Evang. Kliniken Essen-Mitte, Faculty of Medicine, University of Duisburg-Essen, Am Deimelsberg 34 a, 45276 Essen, Germany; a.paul@kem-med.com (A.P.); c.zempel@kem-med.com (C.Z.); h.cramer@kem-med.com (H.C.); g.dobos@kem-med.com (G.D.); a.koch@kem-med.com (A.K.K.); 4 Department of Medical Psychology and Medical Sociology, Ruhr University Bochum, Universitätsstraße 150, 44801 Bochum, Germany; sigrid.elsenbruch@rub.de; 5 National Centre for Naturopathic Medicine, Southern Cross University, Lismore, Lismore Campus, Military Rd, Lismore, NSW 2480, Australia; romy.lauche@scu.edu.au; 6 Technical University of Munich, Chair of Nutrition and Immunology, Gregor-Mendel-Str. 2, 85354 Freising-Weihenstephan, Germany; mohamed.ahmed@tum.de (M.A.); dirk.haller@tum.de (D.H.); 7 ZIEL-Institute for Food and Health, Technical University of Munich, 85354 Freising, Germany

**Keywords:** ulcerative colitis, health-related quality of life, lifestyle-modification, randomized controlled trial, integrative medicine

## Abstract

Patients with ulcerative colitis suffer from impaired health-related quality of life (HrQoL). Comprehensive lifestyle-modification might increase HrQoL and decrease disease activity. Ninety-seven patients in clinical remission with impaired HrQoL were randomly assigned to a 10 week comprehensive lifestyle-modification program (LSM; *n* = 47; 50.28 ± 11.90 years) or control (*n* = 50; 45.54 ± 12.49 years) that received a single workshop of intense training in naturopathic self-help strategies. Primary outcome was HrQoL (Inflammatory Bowel Disease Questionnaire; IBDQ) at week 12. Secondary outcomes included IBDQ subscales; generic HrQoL; disease activity and microbiome. Both groups showed improvement in HrQoL from baseline to post-treatment at week 12. The IBDQ sum score showed no significant group difference (*p* = 0.251). If patients attended more than 50% of the training sessions, a significant group effect (*p* = 0.034) was evident in favor of LSM. In addition, the SF-36 mental component summary (*p* = 0.002) was significantly different between the groups in favor of LSM. Disease activity microbiome and adverse events did not differ. Both a single workshop and a 10-week comprehensive lifestyle-modification program can improve HrQoL in patients with ulcerative colitis in remission with no apparent effects on clinical disease activity. A treatment difference was observed when examining a subsample of patients who attended ≥ 50% of sessions.

## 1. Introduction

Ulcerative colitis represents a chronic inflammatory bowel disease with a high burden of disease for the patients. In Europe, the prevalence is 2.2 million people with a steadily increasing incidence [1]. While the pathogenesis and pathological connections are not fully understood, a connection with the western lifestyle has been suggested, given the higher prevalence in northern Europe and North America [2]. According to the current pathogenetic model, it is assumed that while there is a genetic risk for the disease, the onset and course are significantly influenced by environmental and lifestyle factors [1].

One factor known to contribute to the pathogenesis of ulcerative colitis is psychosocial stress. Seventy percent of patients with ulcerative colitis believe that temporary psychosocial stress may have caused a flare, or at least influenced, the course of their disease in the past [3,4]. In addition, current research gives evidence for bidirectional effects of inflammatory bowel disease (IBD) activity and psychological disorders with promising implications for psychoeducative and psychosocial treatment options in IBD [5]. Furthermore, ulcerative colitis is often associated with reduced quality of life, with a higher level of perceived stress being a strong predictor of patients’ quality of life [6,7]. Quality of life is particularly impaired during flares. In remission, findings are mixed regarding quality of life [8,9]. What is more, in IBD, the reporting of irritable bowel syndrome (IBS)-type symptoms by patients with quiescent disease is common and is associated with psychological disorders, impaired quality of life, and increased health-care use [10]. Our limited understanding of the role of psychosocial and lifestyle factors have hindered the development of effective comprehensive therapeutic approaches. This has further led to patients seeking complementary treatment options outside conventional medication based medicine; often without informing their attending physician [4]. Here, complementary medicine offers a variety of salutogenetic options, with patients reporting benefits beyond improved disease control and beyond effects achieved from using mainly pharmacologic-driven medical approaches; for example, increased quality of life and decreased anxiety [3,11,12].

A comprehensive lifestyle modification program integrates the relevant aspects of complementary medicine (e.g., mind-body medicine, herbal medicine, nutrition, exercise and naturopathic self-help strategies) with the aim of improving patients’ physical, as well as psychological, well-being, in addition to stabilizing the course of disease [13]. Previous research has demonstrated that a multicomponent intervention including mind-body medicine, self-care, stress management strategies, dietary counselling with a focus on the Mediterranean diet, naturopathic self-help strategies and herbal medicine, positively impacts on patients’ quality of life [14,15]. To develop the field, future studies should include patients with higher disease activity and/or higher levels of psychological distress/psychiatric comorbidity [15]. Therefore, within the present study we focused on patients with impaired quality of life and assessed the efficacy of a comprehensive lifestyle modification program on health-related quality of life and disease activity in patients with ulcerative colitis in clinical remission.

## 2. Experimental Section

### 2.1. Study Design

The present study was a prospective randomized controlled trial. Patients were recruited through newspaper and online announcements via a study announcement advertised by the German Crohn’s Colitis Organization, as well as the Department of Internal and Integrative Medicine at the Kliniken Essen-Mitte, Essen, Germany. Prospective participants were first screened via telephone for study eligibility. Eligible patients were then invited to a clinic visit at the Kliniken Essen-Mitte where a study physician provided patients with written information about the study and confirmed eligibility. Eligible patients then gave written informed consent and were included in the study. The study was approved by the Ethics Committee of the University of Duisburg-Essen (approval number 15-6554-BO), registered on clinicaltrials.gov (ID: NCT02721823) and conducted in accordance with the declaration of Helsinki and good clinical practice guidelines.

### 2.2. Study Procedure

The study was conducted at the Kliniken Essen-Mitte from early 2016 (first patient in) until late 2019. There were four consecutive study groups. The first group took part in the study from Feb 2016 to April 2018, the second from August 2016 to September 2018, the third from January 2017 to April 2019, and the fourth from August 2017 to October 2019.

### 2.3. Eligibility Criteria

Patients diagnosed with ulcerative colitis who had been in clinical remission for no longer than12 months at the longest (Clinical Activity Index according to Rachmilewitz (CAI) ≤4 [16]), were aged between 18 and 75 years and had impaired quality of life (Inflammatory Bowel Disease Questionnaire (IBDQ) total score <170 at baseline [17]) were included. Exclusion criteria were: infectious or chronically active colitis; glucocorticosteroids within the last three months (other than stable medication with azathioprine: other pharmaceutical treatments according to the medical guideline were allowed, for example, mesalazine or sulfasalazine); severe psychological illness requiring treatment (e.g., depression, addiction or schizophrenia; severe comorbid somatic disease (e.g., diabetes mellitus or oncological disease)), pregnancy or participation in another stress reduction program or clinical study testing a psychological intervention. In line with current treatment guidelines [1], the inclusion criteria were changed during recruitment to also include patients receiving immunosuppressive medication. Both groups were opened for a stratified inclusion of patients using immunomodulatory medication after the inclusion of 50% of the planned participants.

### 2.4. Randomization

Patients were randomized using stratified block randomization (strata: sex, azathioprine and biologics) to either the comprehensive lifestyle-modification program group or the control group. A biostatistician, not involved in patient recruitment or assessment, generated the random sequence for allocation in a 1:1 ratio (intervention to control) using Random Allocation Software. Using a generated random sequence, the study coordinator concealed the allocation (i.e., intervention or control) within sealed, opaque envelopes by order of ascending number. If a patient met the inclusion and exclusion criteria and agreed to participate, one of the study coordinators (PhD students) opened the envelope with the lowest number and enrolled the patient into the respective group (i.e., intervention or control).

### 2.5. Interventions

#### 2.5.1. Comprehensive Lifestyle-Modification Program

Patients randomized to the comprehensive lifestyle modification group participated in 10 weekly group sessions, each six hours in duration. An experienced team of physicians and mind-body instructors guided the sessions. The day clinic program took place on Thursdays, started at 13:00 with a communal lunch and ended with relaxation therapy at 19:00. At the first appointment, a lecture on the subject of mind-body medicine was given by the principal investigator (author J.L., an experienced gastroenterologist specialized in integrative medicine) and participants were given an overview of the course and objectives of the program. The following nine sessions (sessions 2–10) included theoretical and practical activities on exercise, yoga, stress management, mindfulness, herbal medicines, home remedies, communication, meaning of self-awareness and assessment of personal habits, as well as cooking classes. All sessions were attended by the principal investigator, who gave participants the opportunity to report on their week within a medical round. Patients were also given a variety of information material and were asked to apply what they had learned at home. A detailed description of the program and contents is available via request to the corresponding author.

#### 2.5.2. Control

The control group received a single two-hour psychoeducational workshop, conducted by the principal investigator, on the topic of naturopathic self-care strategies. Various self-care strategies, mind-body techniques, herbal medicines and home remedies, were presented. Patients were also given informational material in the form of a small booklet (KVC Verlag, “Was tun bei Colitis ulcerosa”; Bauchredner 2/16 “Integrative Gesundheit bei chronisch entzündlichen Darmerkrankungen”) which contains general information about the disease, mind-body medicine and self-help. Patients randomized to the control group were offered the option to participate in the comprehensive lifestyle modification program at the end of the study.

### 2.6. Measures

Outcomes were assessed at five different time-points within the lifestyle-modification group: baseline measurement at week 0, postintervention measurement at week 12 and follow-up measurements at weeks 24, 48 and 60. Within the control group, outcomes were assessed at six time-points: baseline measurement at week 0, postintervention measurement at week 12, and follow-up measurements at weeks 24 and 48. After week 48, patients in the control group received the lifestyle modification program; hence the additional postintervention measurement at week 60 and follow-up at week 108. Outcomes were assessed by experienced study nurses, physicians or doctoral students. Sociodemographic and clinical characteristics were captured at baseline. The present manuscript reports on baseline (week 0) and postintervention (week 12) measurements only. Subsequent publications will report on follow-up measurements beyond week 12.

### 2.7. Health-Related Quality of Life

The primary outcome total health-related quality of life at week 12 was assessed using the validated IBDQ German version. [18,19] The IBDQ consists of 32 items with four subscales: bowel (10 items; scale ranging from 10 to 70), systemic (five Items; scale ranging from 5 to 35), emotion (12 items; scale ranging from 12 to 84), and social (five items; scale ranging from 5 to 35), all of which were rated on a 7-point Likert scale (1 = worst rating, 7 = best rating). A total score was then calculated ranging from 32 to 224, with higher scores indicating better health and scores above 170 indicating no impairment to quality of life.

### 2.8. Generic Quality of Life

Generic health-related quality of life was assessed using the 36-Item Short Form Health Survey (SF-36) which is validated and widely used across different health conditions [20,21]. The SF-36 contains eight sub-scores that are weighted sums of the questions for each section: physical functioning (10 items), bodily pain (two items), general health perceptions (five items), physical role limitations (four items), emotional role limitations (three items), social functioning (two items), vitality (four items) and mental health (five items) that were rated on either 5-point Likert scales, 3-point Likert scales or dichotomous items. Higher subscales indicated better health. Furthermore, two component summaries (physical and mental) were calculated using weighted subscales. The eight domains were scored on a 0–100 scale while the two summary measures were norm-based T-scores, with a mean of 50 and SD of 10. In all cases, higher scores indicated better HRQoL.

### 2.9. Clinical Disease Activity

Clinical disease activity was assessed using the Colitis Activity Index (CAI) by Rachmilewitz [16]. The CAI assesses the severity of colitis based on stool frequency, abdominal pain or cramps, blood in stool, extraintestinal manifestations of the disease and laboratory findings. A score higher than 4 indicates a flare; scores of 4 or below currently inactive disease.

### 2.10. Fecal Biomarkers

Inflammatory activity was monitored by noninvasive biomarkers fecal lactoferrin and fecal calprotectin [22]. Stool specimens were collected by the patients and analyzed at an independent laboratory (Labor L+S AG, Bad Bocklet-Großenbrach, Germany). Each specimen was tested for lactoferrin and calprotectin with an enzyme-linked immunosorbent assay (ELISAKits from Immundiagnostik, Bensheim, Germany for calprotectin; IBD-SCAN kit from Techlab, Blacksburg, USA for lactoferrin). 

### 2.11. Endoscopy and Histology

Voluntary endoscopies at weeks 0 and 12 were performed. The presence and degree of active inflammation was quantified using the Endoscopic Score according to Rachmilewitz (EI) [16]. Six mucosal biopsies were taken from rectum and sigma. Biopsies were analyzed and scored by a pathologist, who remained blind to the group allocation throughout the study, using the Riley Score [23].

### 2.12. Clinical Parameters

Medications (Table 1).and blood parameters were assessed during a personal interview which included a physical examination. 

### 2.13. Microbiome

Participants were given a stool collection kit to collect stool specimens. Stool samples were frozen at −80 °C. Analyses were carried out at the Technical University of Munich, Chair of Nutrition and Immunology, Freising, Germany. Bacterial DNA was isolated with an alteration of Godon and colleagues’ method [24]. Basically 500 mg of autoclaved 0.1 mm silica beads (Roth) were added to frozen fecal samples (100–800 mg). Microbial cells were then lysed mechanically (3 × 40 s at 6.5 m/s) using a FastPrep^®^-24 (MP Biomedicals) fitted with a 24 × 2 mL cooling adaptor, heat treated (95 °C, 5 min) and then centrifuged (15,000× *g*, 5 min, 4 °C). Supernatants were then treated with RNase (0.1 μg/μL) for 30 min at 37 °C. Metagenomic DNA was purified using silica membrane-based columns (Macherey-Nagel) following the manufacturer’s recommendations. Genomic DNA concentrations and purity were measured using the NanoDrop^®^ system (Thermo Scientific) and samples were then stored at 4 °C during library preparation, or at −20 °C for longer storage. The V3/V4 region of the 16S ribosomal RNA (rRNA) genes was amplified using polymerase chain reaction (PCR, 25 cycles) from 24 ng of metagenomic DNA using the bacteria-specific primers 341F and 785R [25], followed by a two-step procedure to limit amplification bias [26]. After purification (AMPure XP system, Beckmann) and pooling in an equimolar amount, the V3/V4 regions were sequenced in the paired-end modus (PE275) using an MiSeq device (Illumina, Inc.), as per the manufacturer’s guidelines, and a final DNA concentration of 10 pM and 15% (v/v) PhiX standard library. After sequencing, processed raw data were assigned to their corresponding sample via demultiplexing using previously assigned barcode pairs that were unique to each sample. Afterwards, data were analyzed using the Integrated Microbial Next Generation Sequencing platform [27], which is based on the UPARSE method [28]. For each sample, sequences were dereplicated and checked for chimeras using UCHIME [29]. Sequences from all samples were merged and sorted by abundance, and operational taxonomic units (OTUs) were picked at a threshold of 97% similarity. Finally, all sequences were mapped back to the representative sequences resulting in one OTU table for all samples. Only those OTUs with a relative abundance of above 0.5% total sequences in at least one sample were kept to avoid analysis of spurious OTUs. SILVA (SILVA Incremental Aligner) [30] was used to assign taxonomic classification to the OTUs’ representative sequences. Specific OTUs with differential abundances between groups were further identified using EzTaxon (https://www.ezbiocloud.net/). The OTU table was then refined to the minimum count of sequences observed to prevent incorrect estimation of species richness due to differential sequencing depth. Evaluating beta diversity (diversity between samples) was performed by measuring the distances between microbial profiles using the generalized Unifrac procedure [31]. Beta diversity was visualized by metric multidimensional scaling (MDS) projections of the generalized UniFrac distances. For quantifying alpha-diversity, richness was calculated as the value of present OTUs within one sample. For downstream processing of the intermediate files generated by IMNGS, a fully modular R-based pipeline (Rhea) was used for analysis of microbial profiles [32]. 

### 2.14. Safety

Patients were asked about adverse events at all study visits. Additionally, open-ended questions were used in the questionnaires to assess any adverse events not mentioned to the study team by the patients.

### 2.15. Sample Size Calculation 

In previous studies, a difference of 16 points in the IBDQ was identified as a clinically relevant difference [17,33]. To detect this difference using a two-sample t-test with a 5% significance level and 90% power, and assuming a standard deviation of 23.04 points (as reported for a previous sample [19]), 37 patients per group were needed. Accounting for a maximum dropout rate of 20%, at least 92 patients needed to be enrolled. 

### 2.16. Statistical Analyses 

All analyses were conducted on an intention-to-treat basis, i.e., on all participants randomized irrespective of adherence to the study protocol. Missing values were replaced by multiple imputation methods and 50 additional data sets were generated and averaged. In addition, a per-protocol analysis was performed to explore the impact of adherence to the study protocol on study results. The primary outcome was evaluated using univariate analyses of covariance (ANCOVA) with group as the between-subject factor and baseline values as covariates. Secondary outcomes were evaluated exploratively also using ANCOVA with group as the between-subject factor and baseline values as covariates with no adjusted *p* values for multiple testing. Partial eta-squared (η^2^_p_) was reported as an effect-size estimator. Baseline group differences were analyzed using Student’s t-tests for continuous data and chi-square tests for categorical data. All analyses were performed using the Statistical Package for Social Sciences software (IBM SPSS Statistics for Windows, release 25.0; IBM Corporation, Armonk, NY). A *p*-value <0.05 was considered significant. Microbiome was analyzed using aPermutation Multivariate Analysis of Variance test to determine statistically significant differences between groups for alpha and beta diversity analyses. 

## 3. Results

### 3.1. Patients

**Figure 1:**
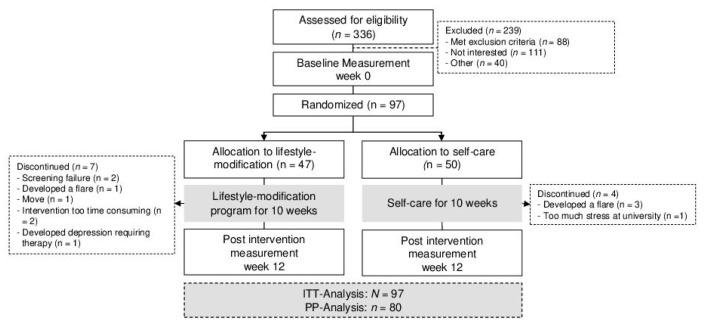
Flow-chart.

336 patients expressed their interest to participate in the study (Figure 1). Ninety-seven patients were invited for further assessment and were all included in the study after providing written informed consent. Patients were randomized to either lifestyle modification (*n* = 47) or control (*n* = 50; Table 1). No baseline differences were evident (all *p* > 0.05) Seven patients in the lifestyle modification group, and four patients in the control, dropped out before week 12. Due to changes between screening and baseline workup at the beginning of the study, patients who (1) had normal IBDQ scores (two in the intervention and two in the control group), or (2) were in remission for more than 12 months (one in the intervention and one in the control group), were included in the study despite violation of the inclusion criteria. Hence, for the per-protocol analyses, 10 patients of the intervention group, and seven patients of the control group, were excluded from the analysis. 

Patients in the lifestyle modification group attended 8.06 ± 2.70 classes on average.

### 3.2. Primary Outcome: Health-Related Quality of Life After 12 Weeks 

**Figure 2:**
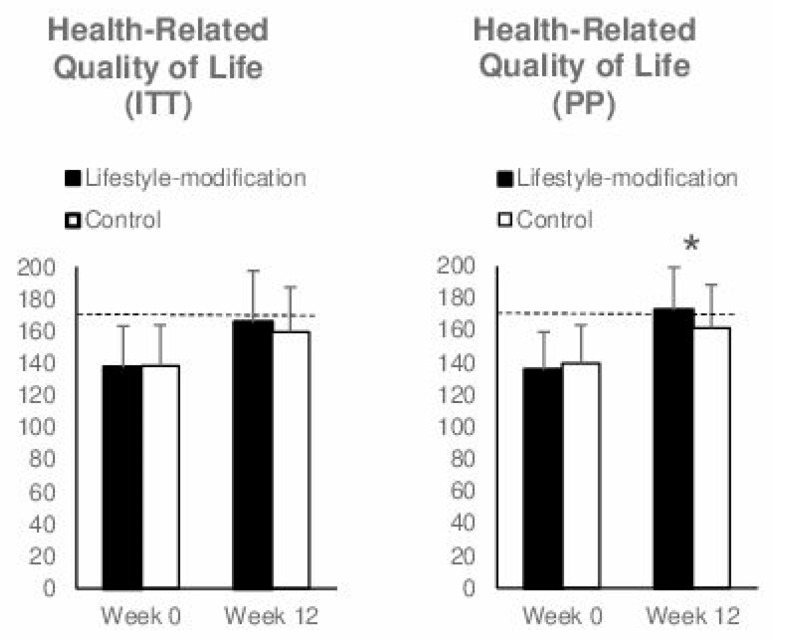
Comprehensive lifestyle-modification program and self-care (control) on health-related quality of life measured with the sum score of the German version of the Inflammatory Bowel Disease Questionnaire. Both groups demonstrated increases in health-related quality of life with no significant difference between the groups within the intention-to-treat analysis (ITT; *N* = 97; *p* = 0.251). Within a per-protocol analysis (PP; *n* = 80), improvement in health-related quality of life was significantly higher in the lifestyle modification group (*p* = 0.034); Values expressed as mean ± standard deviation. Asterisks indicate significant group differences. The dotted line represents the cut-off of 170 indicating no impairment to quality of life.

Patients’ IBDQ total after 12 weeks was not significantly different between groups when analyzing the intention-to-treat sample (F(1, 94) = 1.336, *p* = 0.251, η^2^_p_ = 0.014). After intervention, 40 percent of the control group and 48.94 percent of the intervention group had an IBDQ score above 170. In addition, 52 percent of the controls and 70.21 percent of the intervention group experienced a clinically relevant improvement of 16 points or more. Within the per-protocol analysis, which included 37 patients from the lifestyle modification group and 43 patients from the control group, the IBDQ total score was significantly different between groups at 12 weeks in favor of the lifestyle modification group (F(1, 77) = 4.66, *p* = 0.034, η^2^_p_ = 0.06) (Figure 2). After intervention, 39.53 percent of the control group and 54.05 percent of the intervention group had an IBDQ score above 170. In addition, 51.16 percent of the controls and 75.68 percent of the intervention group experienced a clinically significant improvement of 16 points or more.

### 3.3. Secondary Outcomes

#### 3.3.1. Health-Related Quality of Life Subscales

**Figure 3:**
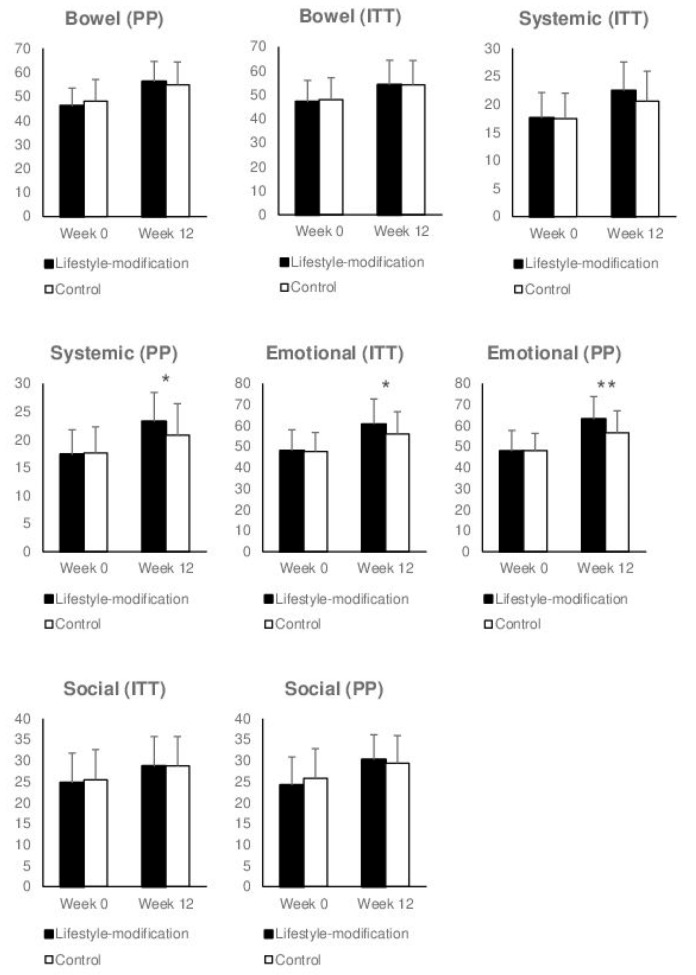
Comprehensive lifestyle-modification program and self-care (control) on subscales of health-related quality of life measured with the German version of the inflammatory bowel disease questionnaire. Within the intention-to-treat-analysis (ITT; *N* = 97), significant group differences in favor of lifestyle-modification were found for the emotional subscale (Emotional (ITT); *p* = 0.045); within the per-protocol analysis (PP; *n* = 80), significant group differences in favor of lifestyle-modification were evident for the emotional (Emotional (PP); *p* = 0.004); and the systemic subscale (Systemic (PP); *p* = 0.034). No further group differences were found (all *p* > 0.05). Values expressed as mean ± standard deviation. Asterisks indicate significant group differences.

Within the intention-to-treat analysis, the IBDQ bowel subscale, systemic subscale and social subscale were not significantly different between the groups. The emotional subscale at week 12 was significantly different between the two groups (F(1, 94) = 4.14, *p* = 0.045, η^2^_p_ = 0.042) in favor of the lifestyle-modification group. Within the per-protocol analysis, the systemic subscale (F(1, 77) = 4.66, *p* = 0.034, η^2^_p_ = 0.057) and the emotional subscale differed between groups at week 12 (F(1, 77) = 8.713, *p* = 0.004, η^2^_p_ = 0.102) in favor of the lifestyle modification group. The bowel and social subscale were not significantly different between groups (Figure 3).

#### 3.3.2. Generic Quality of Life

**Figure 4:**
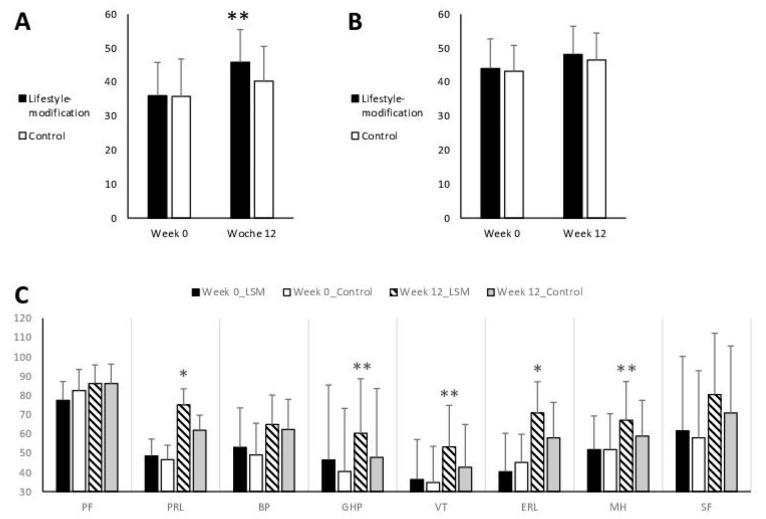
Comprehensive lifestyle-modification program and self-care (control) on generic quality of life measured with the German version of the 36-item Short Form Health Survey, (**A**) mental health index score; (**B**) physical health index score; (**C**) subscales: PF = physical functioning; PRL = physical role limitations; BP = bodily pain; GHP = general health perceptions; VT = vitality; ERL = emotional role limitations; MH = mental health; SF = social functioning. Values expressed as mean ± standard deviation. Asterisks indicate significant group differences.

The SF-36 mental component summary score differed significantly between groups at week 12 (F(1, 94) = 9.820, *p* = 0.002, η^2^_p_ = 0.095) in favor of the lifestyle modification group. The SF-36 physical component summary score did not differ significantly between the two groups. Per-protocol analysis confirmed the differences regarding the SF-36 mental component summary score (F(1, 77) = 11.641, *p* = 0.001, η^2^_p_ = 0.131) but did not reveal significant differences in the SF-36 physical component summary but significant group differences in favor of lifestyle-modification were further evident for physical role limitations (*p* = 0.047), general health perceptions (*p* = 0.003), vitality (*p* = 0.004), emotional role limitations (*p* = 0.026), and mental health (*p* = 0.004; Figure 4).

### 3.4. Disease Activity

There were no significant differences in disease activity (EI, Riley Score, CAI, fecal lactoferrin, or calprotectin; Table 2).

### 3.5. Microbiome Diversity

**Figure 5:**
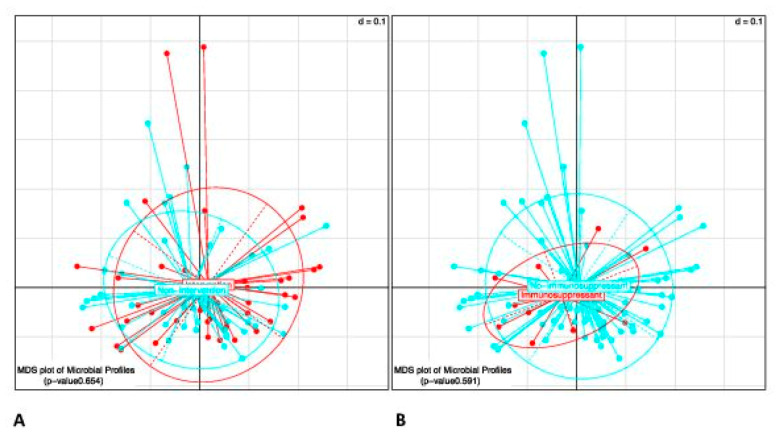
Dimension scaling (MDS) plot shows the generalized unifrac distances of the microbial profiles of the intervention and nonintervention groups at Week 12: No significant difference between the intervention and nonintervention groups at week 12 (**A**) even if controlling for immunosuppressant medications (**B**).

Microbial beta diversity (diversity between samples) was analyzed via generalized UniFrac distance measurement (a distance matrix for microbial communities assessment) and it was visualized by multidimension scaling (MDS). No significant differences were evident between the microbial profiles of the lifestyle-modification and control group at week 12 (Figure 5), even after controlling for immunosuppressant medications. Furthermore, there were no significant differences observed in differential abundance of the operational taxonomic units between the lifestyle-modification and control group. The bacterial communities did not show any noticeable differences at different phylogenetic resolutions (phylum, family and genus).

### 3.6. Safety

Three patients in the lifestyle-modification group and one patient in control group reported one serious adverse event each. The serious adverse event reported in the control group was hospitalization for an acute flare with anemia. Serious adverse events reported in the lifestyle modification group included an abortion with hospitalization, inguinal hernia surgery and surgery for anal stenosis, and were not related to the intervention. Nineteen patients in the lifestyle modification group and twelve patients in the control group reported nonserious adverse events (*p* = 0.188). Nonserious adverse events included, for example, common colds, herpes infection or cystitis.

## 4. Discussion

This paper conveys three messages we believe to be important. Firstly, the results imply that a comprehensive lifestyle-modification program is safe and feasible in patients with ulcerative colitis. Secondly, the program significantly improves health-related quality of life in patients with ulcerative colitis who had mild clinical disease activity and significantly impaired health-related quality of life. Finally, a treatment difference was observed when examining a subsample of patients who attended more than 50% of sessions 

The topic of health-related quality of life is of great importance for patients with ulcerative colitis. Although quality of life impairment is strongly related to disease activity, it is also limited during phases of mild disease activity or even in remission [34,35,36,37]. Therefore, the need to improve the quality of life, and thus relieve the overall disease burden, is very high and oftentimes not part of standard medical treatment [5,10,14]. In the present randomized controlled trial, patients with ulcerative colitis who regularly attended a 10-week comprehensive lifestyle modification program showed significantly better health-related quality of life outcomes after 12 weeks than patients who received a single workshop on naturopathic self-help strategies. Further, compared with controls, comprehensive lifestyle modification improved patients’ emotional symptoms (IBDQ emotional subscale) and, if patients attended regularly, their systemic symptoms. Lifestyle modification, however, had no effect on disease activity (as measured by CAI, fecal lactoferrin fecal calprotectin, EI, and Riley score) in this group of patients with relatively low clinical activity, or even complete clinical remission at baseline. Similarly, no therapy effects on patients’ microbiome were found. Adverse events occurred to a similar extent in both groups.

It was concluded from a previous randomized controlled trial conducted by our group that the effects of a mind-body intervention on ulcerative colitis with mild clinical activity, or in remission, were evident but limited, possibly given the recruitment of a small sample of patients with on average no, or only small, impairments in health-related quality of life [15].Thus, within the present study, recruitment was restricted to patients with existing reduced health-related quality of life. As in the prior study, no effects on disease activity were found. Hence, it may be concluded that multicomponent treatment approaches with a focus on mind-body therapy are effective primarily on measures of quality of life, particularly regarding emotional and systemic symptoms, rather than clinical disease parameters in this group of patients in clinical remission [14]. However, the fact that the included patients were in clinical remission at baseline might support a floor effect. However, interestingly, patients reported that the program had an effect on systemic symptoms of fatigue, malaise and sleep and weight issues, all of which were physical, albeit nonspecific, symptoms. This finding complements the results of Casellas et al. [38]. They described that with inactive disease, systemic symptoms are predominant symptoms that require special attention in therapy. The comprehensive lifestyle-modification program may not have a strong effect on disease activity in colitis patients while in remission, but it may affect nonspecific physical symptoms, thereby alleviating distressing physical conditions of the disease. Future studies should examine this more closely. Further, some effects were significant only in the per-protocol, but not in the intention-to-treat, analysis. Within the per-protocol analysis, only patients who attended at least half of the intervention sessions and did not present as screening failures during the study, were analyzed. These differences in PP analysis were also robust when only those patients with less than 50% participation were excluded from the analysis. Since the control group received a single workshop of intense training in naturopathic self-help strategies, which was also a component of the lifestyle modification program, regular participation in the intervention sessions might have been crucial for the lifestyle modification program to exert its full effects, compared to control. Of note, the control group also showed an improvement in their health-related quality of life after the workshop. One could postulate that the group setting in itself could have had a positive effect beyond that of the actual program content. However, previous studies evaluating the effects of group settings found that group settings are well accepted by patients but do not exert positive effects beyond the actual training content [39,40]. Of course, it is also conceivable that the contents of the workshop itself, as well as positive expectations, had beneficial effects. The comprehensive lifestyle-modification program actively aims to strengthen patients’ coping resources (e.g., social skills, social support and control beliefs). 

During the 10-week program, in addition to treatments specific to ulcerative colitis such as diet, home remedies and herbal medicines, patients also learned generally about health-promoting techniques such as stress management, internal and external communication skills, social network reflection and perception of assessment habits which were intended to strengthen patients’ resources. Although patients were encouraged to incorporate the learned skills into their daily lives, patient compliance was not recorded in this regard. Future studies should address the question of how improvements can be maintained in the long-term; for example, evaluation of nutrition counselling provided. The fact that we could not demonstrate any changes in patients’ microbiomes as a potential indicator of changes in patients’ dietary intake might be caused by an unsuitable time interval to show changes in the microbiome. Moreover, it was hardly possible to calculate the necessary sample sizes. Due to the high interpersonal variability, the required sample size would probably have been very large, so that the size available may not have been sufficient to identify differences [41].

Strengths of the study included the randomized, controlled study design and the assessment of subjective patient-reported outcomes using validated questionnaires in addition to clinical assessments and objective laboratory parameters. The dropout rate was low with only 11.34% attrition after 12 weeks (*n* = 7 in intervention, *n* = 4 in control). There were also limitations in this study. Due to administrative problems at the beginning of the study, the per-protocol analysis was reported in extenso. Furthermore, this study was designed to evaluate changes in total health-related quality of life as measured by the IBDQ post intervention. In addition, study results are limited to a specific group of patients with ulcerative colitis with mild clinical activity or during remission, an interest in complementary treatment approaches and diminished quality of life, which restrict generalizability.

In summary, the study showed that patients with ulcerative colitis might benefit from defined nonpharmacological treatment modules. A comprehensive lifestyle-modification program is safe, feasible and a treatment difference was observed when examining a subsample of patients who attended more than 50% of the training sessions, without effects on disease activity.

## Figures and Tables

**Table 1:** Sociodemographic and clinical characteristics at baseline. Values are expressed as mean ± standard deviation, unless indicated otherwise.

	Lifestyle-Modification (*n* = 47)	Control (*n* = 50)
Age years	50.28 ± 11.90 (18–74)	45.54 ± 12.49 (19–71)
Female *n* (%)	34 (72.3)	35 (70)
Weight	72.79 ± 14.90 (52–100)	70.24 ± 16.86 (49.6–150)
Height	171.19 ± 9.05 (152–196)	173.76 ± 9.94 (156–197)
Anamnestic pattern *n* (%)		
Proctitis	14 (29.8)	15 (30)
Left-sided colitis	17 (36.2)	15 (30)
Pancolitis	13 (27.7)	17 (34)
Missing	3 (6.4)	3 (6)
Time since diagnosis in years	18.04 ± 12.00 (2–46)	14.76 ± 10.99 (1–43)
Prior integrative medicine inpatient treatment at Kliniken Essen-Mitte *n* (%)	13 (27.7)	12 (24)
Prior integrative medicine day-care treatment at Kliniken Essen-Mitte *n* (%)	7 (14.9)	3 (6)
Smokers *n* (%)	2 (4.3)	3 (6)
Married *n* (%)	33 (70.2)	39 (78)
Education *n* (%)		
Secondary school	17 (36.1)	11 (22)
High school (“Abitur”)	12 (25.6)	14 (28)
University degree	18 (38.3)	25 (50)
Blood parameters		
Leucocytes	6.40 ± 1.70	6.73 ± 4.38
Thrombocytes	272.26 ± 81.69	269.98 ± 72.68
Blood sedimentation rate	9.17 ± 10.55	9.54 ± 11.99
C-reactive protein	0.36 ± 0.67	0.29 ± 0.58
Medication intake *n* (%)		
Steroids	2 (4.3)	1 (2)
Azathioprine	4 (8.5)	3 (6)
Mesalazine	33 (70.2)	34 (68)
Herbal medicine	7 (14.9)	15 (30)
Biologicals	3 (6.4)	3 (6)
Other	8 (17)	12 (24)

**Table 2:** Disease activity as measured by the clinical activity index, fecal lactoferrin, and fecal calprotectin. Values are expressed as median (minimum-maximum), unless indicated otherwise.

	*n*	Baseline	Week 12	Group Differences	
				*p*	η^2^_p_
Rachmilewitz Endoscopic Score (M ± SD)					
Lifestyle-modification ITT	15	2.47 ± 2.77	2.73 ± 2.49	0.451	0.020
Control ITT	16	2.19 ± 2.48	3.13 ± 3.20		
Lifestyle-modification PP	13	2.62 ± 2.96	2.62 ± 2.47	0.227	0.063
Control PP	13	2.62 ± 2.57	3.69 ± 3.30		
Riley Score (M ± SD)					
Lifestyle-modification ITT	15	4.89 ± 4.55	5.53 ± 4.96	0.406	0.025
Control ITT	16	5.00 ± 4.23	4.31 ± 4.81		
Lifestyle-modification PP	13	5.14 ± 4.78	5.77 ± 5.25	0.586	0.013
Control PP	13	5.77 ± 4.34	5.08 ± 5.04		
CAI (M ± SD)					
Lifestyle-modification ITT	47	2.30 ± 1.21	1.74 ± 1.78	0.239	0.015
Control ITT	50	2.12 ± 1.27	2.12 ± 2.00		
Lifestyle-modification PP	37	2.38 ± 1.11	1.65 ± 1.93	0.179	0.023
Control PP	43	2.19 ± 1.34	2.19 ± 2.12		
Fecal lactoferrin					
Lifestyle-modification ITT	45	3.85 (0.13–97.47)	8.42 (0.12–61.96)	0.648	0.002
Control ITT	48	3.83 (0.08–85.16)	4.03 (0.08–60.79)		
Lifestyle-modification PP	37	4.97 (0.13–97.47)	4.53 (0.12–61.69)	0.510	0.006
Control PP	43	3.47 (0.08–85.16)	3.91 (0.08–60.79)		
Fecal calprotectin					
Lifestyle-modification ITT	45	100.59 (2.48–1375.50)	80.81 (10.48–1232.40)	0.751	0.001
Control ITT	48	99.49 (6.92–1900.10)	95.53 (28.51–1660.50)		
Lifestyle-modification PP	37	114.32 (2.48–1375.50)	75.15 (10.48–1232.40)	0.855	0.000
Control PP	43	101.53 (6.92–1900.10)	95.43 (28.51–1660.50)		

Note. *p*-values are based on univariate analyses of covariance with group as the between-subject factor and baseline values as covariates. CAI = clinical activity index; M = mean; SD = standard deviation; ITT = intention-to-treat analysis; PP = per-protocol analysis.
